# Revising the self-report strengths and difficulties questionnaire for cross-country comparisons of adolescent mental health problems: the SDQ-R

**DOI:** 10.1017/S2045796019000246

**Published:** 2019-05-03

**Authors:** E. L. Duinhof, K. M. Lek, M. E. de Looze, A. Cosma, J. Mazur, I. Gobina, A. Wüstner, W. A. M. Vollebergh, G. W. J. M. Stevens

**Affiliations:** 1 Department of Interdisciplinary Social Science, Faculty of Social and Behavioural Sciences, Utrecht University, P.O. Box 80.140, 3508 TC Utrecht, The Netherlands; 2 Department Methodology and Statistics, Faculty of Social and Behavioural Sciences, Utrecht University, P.O. Box 80.140, 3508 TC Utrecht, The Netherlands; 3 Department of Psychology, Babes Bolyai University, Cluj Napoca, Republicii Street 37, 400015, Romania; 4 Department of Child and Adolescent Health, Institute of Mother and Child, 01-211 Warsaw, Kasprzaka 17a str, Poland; 5 Department of Public Health and Epidemiology, Faculty of Public Health and Social Welfare and Institute of Public Health, Rīga Stradiņš University, Rīga, Kronvalda bulv. 9, Latvia; 6 Department of Child and Adolescent Psychiatry, Psychotherapy and Psychosomatics, University Medical Center Hamburg-Eppendorf, Martinistr. 52, 20246 Hamburg, Germany

**Keywords:** Adolescents, epidemiology, mental health, minority issues and cross cultural psychiatry, validation study

## Abstract

**Aims:**

The Strengths and Difficulties Questionnaire (SDQ) has been used in many epidemiological studies to assess adolescent mental health problems, but cross-country comparisons of the self-report SDQ are scarce and so far failed to find a good-fitting, common, invariant measurement model across countries. The present study aims to evaluate and establish a version of the self-report SDQ that allows for a valid cross-country comparison of adolescent self-reported mental health problems.

**Methods:**

Using the Health Behaviour in School-aged Children study, the measurement model and measurement invariance of the 20 items of the self-report SDQ measuring adolescent mental health problems were evaluated. Nationally representative samples of 11-, 13- and 15-year old adolescents (*n* = 33 233) from seven countries of different regions in Europe (Bulgaria, Germany, Greece, the Netherlands, Poland, Romania, Slovenia) were used.

**Results:**

In order to establish a good-fitting and common measurement model, the five reverse worded items of the self-report SDQ had to be removed. Using this revised version of the self-report SDQ, the SDQ-R, partial measurement invariance was established, indicating that latent factor means assessing conduct problems, emotional symptoms, peer relationships problems and hyperactivity-inattention problems could be validly compared across the countries in this study. Results showed that adolescents in Greece scored relatively low on almost all problem subscales, whereas adolescents in Poland scored relatively high on almost all problem subscales. Adolescents in the Netherlands reported the most divergent profile of mental health problems with the lowest levels of conduct problems, low levels of emotional symptoms and peer relationship problems, but the highest levels of hyperactivity-inattention problems.

**Conclusions:**

With six factor loadings being non-invariant, partial measurement invariance was established, indicating that the 15-item SDQ-R could be used in our cross-country comparison of adolescent mental health problems. To move the field of internationally comparative research on adolescent mental health forward, studies should test the applicability of the SDQ-R in other countries in- and outside Europe, continue to develop the SDQ-R as a cross-country invariant measure of adolescent mental health, and examine explanations for the found country differences in adolescent mental health problems.

## Introduction

Worldwide, a significant percentage of adolescents experience mental health problems (Polanczyk *et al*., 2015). As these problems are likely to continue into adulthood (Rutter *et al*., 2006), mental health promotion efforts in adolescence are a global public health priority (Patel *et al*., 2007). To advance population-based knowledge of adolescent mental health, cross-country comparisons are essential (Achenbach *et al*., 2012). There is clear evidence of cross-country variation in adolescent *subjective well-being* (e.g., life satisfaction) in Europe (Bradshaw and Richardson, 2009; Klocke *et al*., 2014; Inchley *et al*., 2016), but global prevalence data on adolescent *mental health problems* are scarce (Erskine *et al*., 2017).

The Strengths and Difficulties Questionnaire (SDQ) (Goodman, 1997) is one of the most frequently used instruments to assess mental health problems (i.e., emotional, behavioural and relational problems) in adolescents. It has been included in epidemiological studies in various *individual* countries to assess population levels of adolescent mental health problems (see http://www.sdqinfo.org). However, *cross-country comparisons* based on the self-report SDQ are scarce and faced methodological challenges which are lined out below (Ravens-Sieberer *et al*., 2008; Essau *et al*., 2012; Ortuño-Sierra *et al*., 2015; Stevanovic *et al*., 2015; De Vries *et al*., 2018).

First, samples that are compared should be nationally representative and sample characteristics, sampling methods and data collection methods should be comparable across countries (Achenbach *et al*., 2012). However, this is often not the case in the available cross-country literature. Of the few cross-country studies that used the self-report SDQ, some only included adolescents from specific regions within countries (Essau *et al*., 2012; Stevanovic *et al*., 2015), they compared national samples with different gender or age distributions (Essau *et al*., 2012; Ortuño-Sierra *et al*., 2015; Stevanovic *et al*., 2015), or they compared national samples that were collected with different sampling methods (i.e., school- *v*. household-based surveys) (Ravens-Sieberer *et al*., 2008), or data collection methods (e.g., collective or individual questionnaire administration) (Ravens-Sieberer *et al*., 2008; Ortuño-Sierra *et al*., 2015), that each may impact estimates of adolescent mental health problems (e.g., Vollebergh *et al*., 2006). Thus, it is not clear whether the cross-country variation observed in these studies reflect actual or methodological differences in adolescent mental health problems between countries (Achenbach *et al*., 2012).

Second, to make valid comparisons, studies should test whether the structure of the underlying constructs measured by the SDQ (a common measurement model), and the meanings ascribed to these underlying constructs (measurement invariance) are comparable across countries. Only some of the cross-country studies on the self-report SDQ tested the (meaning of the) underlying constructs of the SDQ. These studies either did not find a common measurement model across different countries (Stevanovic *et al*., 2015), or had to allow correlated residuals between items (Ortuño-Sierra *et al*., 2015) to establish a common measurement model. Such modifications may however not replicate in different data sets (Kyriazos, 2018). Often, model fit issues were related to the five reverse worded items of the SDQ: they cross-loaded on the prosocial behaviour subscale or negatively affected the overall model fit (Essau *et al*., 2012; Ortuño-Sierra *et al*., 2015). Those studies that did establish a common measurement model did not find evidence for measurement invariance (Essau *et al*., 2012) or established partial measurement invariance (Ortuño-Sierra *et al*., 2015).

Because of these challenges, it has been argued that the self-report SDQ in its present form is not suitable for cross-country comparisons (Stevanovic *et al*., 2017) and needs to be revised (Essau *et al*., 2012; Ortuño-Sierra *et al*., 2015; Stevanovic *et al*., 2015). More specifically, it has been suggested that the reverse worded items of the SDQ should be re-worded or removed (Essau *et al*., 2012). Also, it has been argued that the measurement model should be examined in countries across different regions in- and outside Europe (Ortuño-Sierra *et al*., 2015; De Vries *et al*., 2018).

The present study sets out to evaluate and establish a version of the self-report SDQ that can be used to validly compare mental health problems of 11-, 13- and 15-year old adolescents across seven European countries. We attempt to overcome the former methodological challenges by (1) using national representative samples of adolescents with similar sample characteristics, assessed with similar sampling and data collection methods in seven countries of different regions in Europe (Bulgaria, Germany, Greece, the Netherlands, Poland, Romania, Slovenia), (2) establishing a good-fitting, common measurement model using cross-validation to assess the replicability of model modifications, and (3) testing the invariance of this common measurement model.

## Methods

### Participants

Data on the self-report SDQ from the Health Behaviour in School-aged Children (HBSC) study that were collected in the 2005/2006 (Poland), 2009/2010 (Germany, Greece) and 2013/2014 (Bulgaria, the Netherlands, Slovenia, Romania) surveys were used. HBSC is a cross-sectional, school-based survey that is conducted every 4 years across more than 40 countries in Europe and North America. Using a standardised research protocol, self-report questionnaires are administered to nationally representative samples of 11-, 13- and 15-year-olds in the classroom. Samples are drawn using cluster sampling, with schools or school classes as primary sampling units. School response rates varied by country but were >80% in all countries except in the Netherlands (49%). At the student-participant level, response rates ranged from 78 to 94%. More information can be found elsewhere (Currie *et al*., 2010, 2014).

In the Netherlands (2005/2006, 2009/2010, 2013/2014) and Bulgaria (2005/2006, 2013/2014) self-report SDQ data were collected in *multiple* HBSC surveys. Results showing that the measurement model of the SDQ is invariant across these timepoints in the Netherlands (Duinhof *et al*., 2015) and Bulgaria (Appendix A), justify the inclusion of only the most recent 2013/2014 data for the Netherlands and Bulgaria. We merge the 2013/2014 data of the Netherlands and Bulgaria with the 2005/2006, 2009/2010 and 2013/2014 data of the other countries, assuming that in these countries the self-report SDQ would be invariant over different timepoints as well.

The total sample consisted of 33 233 11-, 13- and 15-year old adolescents, 51% were girls (ranging between 47.7 and 53.3% across countries). No significant (*p* > 0.001) gender and age distribution differences were found across the country samples. Adolescents who did not fill in the SDQ (*n* = 279, 0.8% of the total sample) were excluded from the analyses. For the remaining samples, missing item responses ranged from 0.1 to 3.3%.

### Measures

Adolescents filled in the self-report SDQ (Goodman, 1997) in their national language. The self-report SDQ is a 25-item questionnaire for 11–17 year olds. It consists of four subscales measuring mental health problems (conduct problems, emotional symptoms, peer relationship problems, hyperactivity-inattention problems) and one subscale measuring strengths (prosocial behaviour). In the present study, data were only available for the problem subscales. Each subscale comprises five items that are scored on a three-point ordinal Likert scale (0 = ‘Not true’, 1 = ‘Somewhat true’, 2 = ‘Certainly true’). Items are phrased in the direction of their subscales, with higher scores indicating higher problem levels, except for five reverse worded items: obedient, has good friend, generally liked, thinks before acting and good attention. The exact wording of the items and abbreviations used in this study can be found in Appendix B.

Adolescents indicated their gender by responding to the question: ‘Are you a boy or a girl?’. Age was determined based upon the participant's month and year of birth and the date of survey administration.

### Analytical strategy

Analyses were performed in Mplus 8.2 (Muthén and Muthén, 2017), using the weighted least squares mean and variance adjusted estimator and the theta parameterisation. Analyses were corrected for cluster effects of adolescents in the same school.

#### Step 1: Establishing a common measurement model

To establish a common measurement model we collated the data from all countries. A common measurement model was *only* established if the model showed an acceptable to good fit in this total sample and in each individual country. Based on preliminary analyses (see Appendix C) and findings from previous cross-country comparisons supporting a first-order five-factor model (Essau *et al*., 2012; Ortuño-Sierra *et al*., 2015), a first-order model with four correlated factors measuring mental health problems was used as a starting point.

Using confirmatory factor analysis (CFA), a common measurement model was established considering the following guidelines. First, to find a parsimonious common measurement model that corresponds to the theoretical structure introduced by Goodman (1997) *and* to protect against non-theory-driven model modifications that might not replicate in other samples (Hermida, 2015; Kyriazos, 2018), correlated item residuals and cross-loadings of items were not allowed. Second, items with non-significant factor loadings and/or standardised factor loadings below 0.40 were considered unacceptable (Ford *et al*., 1986). When supported by previous empirical findings, these items were removed. Third, the overall model fit was evaluated (acceptable fit = RMSEA <0.08 and CFI >0.90; good fit = RMSEA <0.05, CFI >0.96) (Browne and Cudeck, 1992; Hu and Bentler, 1999; Yu, 2002). If models did not show acceptable fits, model modification indices (MI) were consulted to review misspecified model parameters. As MI may be driven by characteristics of the sample on which the measurement model is tested (Byrne, 2012), cross-validation was used. Of the total sample, 9/10th was used to test and modify models using MI while a random 1/10th was used for validation purposes. Only if a good fitting model was established in both the test and validation set, validation was ended.

Internal consistency of the problem subscales was examined as a quality indicator of the final common measurement model using the ordinal alpha coefficient. Ordinal alpha values above 0.70 were considered acceptable (Nunnally and Bernstein, 1967; Gadermann *et al*., 2012).

#### Step 2: Invariance testing

To make valid cross-country comparisons, a common measurement model should be established (configural invariance), items should have invariant relationships to their latent factors across countries (metric invariance) and adolescents in different countries should report invariant average scores on the items (scalar invariance). The three-step method testing configural, metric and scalar models was used. First, a configural model with factor loadings and thresholds freely estimated across countries was tested. Second, a metric model with factor loadings constrained equal across countries was examined. Third, a scalar model with factor loadings and thresholds constrained equal across countries was tested.

If invariance tests indicated a lack of metric or scalar invariance, partial invariance can be established and latent means scores can still be compared across countries (Steinmetz, 2013; Bowen and Masa, 2015). Partial measurement invariance was established by freeing the factor loading/threshold of one item at the time, starting with the factor loading/threshold with the highest MI (Dimitrov, 2010). Our analyses showed that only MI accompanied by a meaningful expected parameter change increased model fit. Hence, both values were inspected to identify non-invariant item factor loadings/thresholds. Changes in CFI values (ΔCFI ⩾ −0.010) and RMSEA values (ΔRMSEA ⩾ 0.015) compared to the configural or metric model were used to evaluate whether (partial) invariance criteria were met (Cheung and Rensvold, 2002; Chen, 2007). Following Dimitrov (2010), partial measurement invariance was established if <20% of the factor loadings and thresholds were non-invariant across *all* countries.

#### Step 3: Cross-country comparisons

If (partial) measurement invariance was established, latent means were compared across countries. Since significant latent mean differences are easy to find in large samples, we applied a stringent significance level (*p* < 0.001) and examined the substantially of the latent mean differences by evaluating the size of the *standardised* latent mean differences using Cohen's *d* (Cohen, 1988). In multi-group CFA, Mplus by default fixes the means of the latent variables in the first group to zero. Bulgaria was arbitrarily set as the reference country.

## Results

### Step 1: Establishing a common measurement model

Table 1 shows the fit indices of the models tested to establish a common measurement model. Models testing and validating the first-order four-factor model failed to demonstrate acceptable CFI values (Table 1; Model 1 and 2). The reverse worded item *obedient* was not related to the conduct problem subscale in both the first (*β* = 0.01, *p* = 0.52, *R*^2^ = 0.00), and second model (*β* = −0.03, *p* = 0.17, *R*^2^ = 0.00). Standardised factor loadings of the other reverse worded items belonging to the peer relationship problems and hyperactivity-inattention problems subscales were unsatisfactory low with standardised factor loadings below 0.40. Only in the validation model *good attention* loaded just satisfactory on the hyperactivity-inattention problems subscale (*β* = −0.41, *R*^2^ = 0.17).

**Table 1.** Fit indices of the models tested to establish a common measurement model

Models	*n*^a^	*χ*^2^	df	CFI	RMSEA
Total sample					
1. Basic model	29 910	17 905.45*	164	0.816	0.060
2. Validation of model 1	3323	2258.08*	164	0.850	0.062
3. Remove obeys	29 903	14 635.08*	146	0.849	0.058
4. Validation of model 3	3323	2319.08*	146	0.845	0.067
5. Remove other reverse worded items	29 896	5763.07*	84	0.933	0.048
6. Validation of model 5	3324	747.96*	84	0.942	0.049
7. Final common measurement model	33 220	6336.69*	84	0.931	0.047
Individual countries					
Bulgaria	4712	1407.32*	84	0.946	0.058
Germany	4991	888.10*	84	0.932	0.044
Greece	4910	979.01*	84	0.937	0.047
Netherlands	4241	864.92*	84	0.945	0.047
Poland	5486	1259.26*	84	0.936	0.051
Romania	3886	1078.16*	84	0.899	0.055
Slovenia	4994	2298.02*	84	0.903	0.073

*Note.* * = *p* < 0.001.

a 7 adolescents in Bulgaria, 5 adolescents in Romania, and 1 adolescent in Greece had missing values on all SDQ items of the final common measurement model and were excluded from the analysis.

To increase model fit, the non-significant item *obedient* was removed. Model 3 and 4 show that after removing this item CFI values remained unacceptably low. Similar to Model 1 and 2, factor loadings of the remaining reverse worded items were unsatisfactorily low (*β* < 0.40), and only a small proportion of their variance was explained by their corresponding latent factors (*R*^2^ range = 0.08–0.13). The MIs of Model 3 and 4 also indicated problems with the reverse worded items. In both models, the two largest MIs suggested to correlate the residuals of the reverse worded items belonging to the same subscale (peer relationship problems or hyperactivity-inattention problems). Given these findings, our aim to establish a parsimonious common measurement model that replicates in future studies, and the numerous studies indicating problems with the reverse worded items (e.g., Essau *et al*., 2012; Ortuño-Sierra *et al*., 2015), we decided to remove the remaining reverse worded items. This resulted in a good model fit (Table 1; Model 5 and 6), with CFI values nearing 0.96 and RMSEA values below 0.05. Therefore we took this model without the reverse worded items as the final measurement model and tested its fit on the total sample and in each individual country. The final common measurement model showed a good fit on the total sample (Table 1; Model 7) and acceptable factor loadings were found for all items (*β* > 0.40) (Table 4). The final measurement model reached an acceptable to good fit in Bulgaria, the Netherlands, Germany, Greece and Poland, with CFI values near 0.96 and RMSEA values below or close to 0.05, and an acceptable model fit in Slovenia and Romania, with CFI values of or above 0.90, and RMSEA below 0.08 (Table 1). Except for the items *steals* (*β* = 0.33) and *prefers adult* (*β* = 0.34) in Poland, in all countries, items loaded satisfactorily on their latent factors (*β* > 0.40). Table 2 shows that in all countries, the emotional symptoms and hyperactivity-inattention subscales showed acceptable internal consistencies (*α* close to or above 0.70). The conduct problems subscale showed acceptable internal consistencies in most countries, with Greece and Slovenia reporting ordinal *α* values slightly below 0.70. Only in Poland, an unsatisfactorily low ordinal *α* value was found for the conduct problem subscale (*α* = 0.60). In all countries, the peer relationship problems subscale had a low internal consistency.

**Table 2.** Ordinal alpha values of the problem subscales in each country

Country	Conduct problems	Emotional symptoms	Peer relationship problems	Hyperactivity-inattention problems
Bulgaria	0.70	0.82	0.65	0.69
Germany	0.72	0.78	0.55	0.71
Greece	0.66	0.79	0.60	0.70
Netherlands	0.72	0.81	0.59	0.81
Poland	0.60	0.76	0.55	0.74
Romania	0.71	0.79	0.58	0.68
Slovenia	0.68	0.82	0.61	0.77

### Step 2: Invariance testing

Measurement invariance was tested across countries (Table 3). The configural model (i.e., the common measurement model), with no equality constraints across countries, showed an acceptable fit to the data. Constraining factor loadings equal across countries decreased the model fit (ΔCFI ⩾ 0.010), showing that latent factors had no equivalent relationships with *all* items across countries and that metric invariance was not supported. After the factor loadings of six items were set free in specific countries (see footnote Table 3), partial metric invariance was established. After establishing partial metric invariance, we tested for scalar invariance. Scalar invariance was found (ΔCFI = −0.006). With six-factor loadings being non-invariant of the total 45 parameters in the measurement model (i.e., 15 factor loadings and 30 thresholds), the observed percentage of non-invariance across all countries was 13.3%. The resulting final partially invariant model showed an acceptable fit to the data (Table 3; Model 4).

**Table 3.** Fit indices of the models testing for invariance across countries

Models	*χ*^2^	df	CFI	RMSEA	ΔCFI	ΔRMSEA	Model comparisons
1. Configural	8619.29*	588	0.929	0.054	–	–	–
2. Metric	10 862.83*	654	0.910	0.057	−0.019	0.003	2 *v*. 1
3. Partial Metric^a^	9733.16*	644	0.920	0.055	−0.009	0.001	3 *v*. 1
4. Scalar	10 425.72*	710	0.914	0.054	−0.006	−0.001	4 *v*. 3

*Note*. * = *p* < 0.001

a Factor loadings of *fights* in Greece and Slovenia, *lies* in Greece and the Netherlands, *clingy* in the Netherlands, *prefers adult* in Poland and the Netherlands, *fidgety* in Greece and Germany and *distractible* in Romania set free.

Table 4 shows that in the final partially invariant model all items loaded satisfactorily on their latent factors (*β*s > 0.40). Only in Poland the *prefers adult* item loaded unsatisfactorily low (*β* = 0.33) on the peer relationship problems subscale and the *fights* and *steals* items loaded just satisfactorily (*β*s = 0.40/0.41) on the conduct problems subscale. The final model included a warning about the high correlations between the latent factors in Romania. The results of Table 4 support this warning, especially the correlation between peer relationship problems and conduct problems is exceptionally high in Romania (*r* = 0.98). In general, latent factor intercorrelations were high (see Table 4), indicating that models with less factors might be a better fit to the data. However, additional CFA analyses testing a one-factor solution (measuring general mental health problems) and a two-factor solution (measuring internalising and externalising mental health problems) did not support this (see Appendix D).

**Table 4.** Fully standardised factor loadings and latent factor correlations of the final common measurement model and the final partially invariant model

Problem subscales	Fully standardised factor loadings (*R*^2^)							
	Common measurement model	Bulgaria	Germany	Greece	Netherlands	Poland	Romania	Slovenia
Conduct problems								
Tempers	0.67 (0.45)	0.67 (0.45)	0.71 (0.51)	0.68 (0.46)	0.77 (0.59)	0.68 (0.46)	0.68 (0.47)	0.71 (0.50)
Fights	0.50 (0.25)	0.50 (0.25)	0.56 (0.31)	0.57 (0.33)	0.48 (0.23)	0.40 (0.16)	0.56 (0.31)	0.52 (0.27)
Lies	0.63 (0.40)	0.64 (0.41)	0.66 (0.44)	0.52 (0.27)	0.69 (0.47)	0.53 (0.28)	0.66 (0.43)	0.60 (0.36)
Steals	0.51 (0.26)	0.57 (0.32)	0.54 (0.29)	0.55 (0.30)	0.46 (0.21)	0.41 (0.17)	0.54 (0.29)	0.51 (0.26)
Emotional symptoms								
Somatic symptoms	0.56 (0.32)	0.62 (0.39)	0.51 (0.26)	0.59 (0.35)	0.53 (0.28)	0.51 (0.26)	0.63 (0.40)	0.57 (0.33)
Worries	0.68 (0.46)	0.73 (0.53)	0.62 (0.39)	0.66 (0.44)	0.66 (0.44)	0.71 (0.51)	0.64 (0.41)	0.76 (0.58)
Unhappy	0.80 (0.64)	0.79 (0.62)	0.82 (0.67)	0.75 (0.57)	0.88 (0.77)	0.79 (0.63)	0.80 (0.65)	0.82 (0.67)
Clingy	0.65 (0.43)	0.73 (0.53)	0.63 (0.40)	0.70 (0.49)	0.66 (0.44)	0.61 (0.37)	0.61 (0.37)	0.70 (0.49)
Fears	0.60 (0.36)	0.58 (0.34)	0.65 (0.42)	0.59 (0.35)	0.65 (0.43)	0.54 (0.29)	0.57 (0.32)	0.61 (0.38)
Peer relationship problems								
Solitary	0.59 (0.34)	0.74 (0.55)	0.50 (0.25)	0.64 (0.41)	0.49 (0.24)	0.69 (0.48)	0.61 (0.37)	0.57 (0.33)
Bullied	0.67 (0.45)	0.70 (0.49)	0.65 (0.42)	0.66 (0.43)	0.72 (0.51)	0.62 (0.38)	0.63 (0.40)	0.74 (0.55)
Prefers adult	0.44 (0.19)	0.42 (0.17)	0.46 (0.21)	0.46 (0.21)	0.51 (0.26)	0.33 (0.11)	0.44 (0.19)	0.46 (0.21)
Hyperactivity-inattention problems								
Restless	0.67 (0.45)	0.69 (0.48)	0.76 (0.58)	0.61 (0.38)	0.77 (0.59)	0.66 (0.44)	0.55 (0.30)	0.75 (0.57)
Fidgety	0.71 (0.51)	0.61 (0.37)	0.59 (0.35)	0.75 (0.56)	0.81 (0.66)	0.78 (0.60)	0.67 (0.45)	0.87 (0.75)
Distractible	0.71 (0.50)	0.65 (0.43)	0.70 (0.49)	0.64 (0.40)	0.72 (0.52)	0.69 (0.48)	0.74 (0.55)	0.76 (0.58)
Latent factor correlations								
EMO with COND	0.72	0.87	0.52	0.71	0.59	0.82	0.82	0.74
PEER with COND	0.80	0.88	0.70	0.75	0.70	0.71	0.98	0.82
PEER with EMO	0.77	0.83	0.69	0.79	0.67	0.79	0.87	0.72
HYP with COND	0.72	0.86	0.60	0.90	0.60	0.85	0.88	0.69
HYP with EMO	0.64	0.89	0.40	0.71	0.44	0.75	0.76	0.61
HYP with PEER	0.48	0.63	0.29	0.59	0.25	0.53	0.68	0.45

*Note*. All factor loadings, explained variance (*R*^2^), and correlations between latent factors were significant at *p* < 0.001.

EMO, emotional symptoms; COND, conduct problems; HYP, hyperactivity–inattention problems; PEER, peer relationship problems.

### Step 3: Cross-country comparisons

To describe cross-country differences in adolescent mental health problems, Table 5 displays country rankings for each problem subscale based on the *unstandardised* latent mean differences, with Bulgaria as the reference country. Higher rankings indicate higher latent mean levels of adolescents' self-reported problems. Setting other countries as the reference country resulted in similar rankings. To evaluate the substantiality of these cross-country differences, Table 5 also includes *standardised* latent mean differences (*d*). Only significant (*p* < 0.001) *and* substantial (*d* > 0.20) latent mean differences were considered indicative of cross-country differences. Adolescents in Poland reported the highest levels of emotional symptoms and conduct problems. Adolescents in Greece reported the lowest levels of emotional symptoms (together with adolescents in Bulgaria), peer relationship problems and hyperactivity-inattention problems. Adolescents in Bulgaria, Germany and Slovenia reported the highest levels of peer relationship problems. Adolescents in the Netherlands reported the lowest levels of conduct problems, but the highest levels of hyperactivity-inattention problems.

**Table 5.** Cross-country rankings based on unstandardised latent mean differences and standardised latent mean differences (*d*) across countries

Country	Conduct problems		Emotional symptoms		Peer relationship problems		Hyperactivity-inattention problems	
	Ranks	*d*	Ranks	*d*	Ranks	*d*	Ranks	*d*
Bulgaria	4	0	1	0	6	0	4	0
Germany	2	−0.41*	5	0.30*	7	0.06	5	0.11
Greece	6	0.13	2	0.07	1	−0.64*	1	−0.26*
Netherlands	1	−0.61*	3	0.22*	2	−0.31*	7	0.36*
Poland	7	0.33*	7	0.54*	4	−0.23*	3	−0.10
Romania	3	−0.25*	4	0.24*	3	−0.24*	2	−0.12
Slovenia	5	0.12	6	0.34*	5	−0.02	6	0.09

*Note*.* = *p* < 0.001; Higher rankings indicate higher mean levels of problems.

## Discussion

By applying cross-validation and using nationally representative samples of seven countries of different European regions assessed with similar sampling and data collection methods, this study established a revised version for the problem subscales of the self-report SDQ (SDQ-R). To construct this good-fitting, common measurement model, the five reverse worded items of the self-report SDQ had to be removed. The SDQ-R was found to have a *sufficient* amount of invariant items, indicating that adolescent mental health problems could be validly compared across the seven countries in this study. By establishing the SDQ-R, this study contributes to the scarce literature on the cross-cultural validity of scales that examine adolescent mental health problems (Stevanovic *et al*., 2017).

Our findings are in line with previous internationally comparative studies, that also indicated problems with the five reverse worded items of the SDQ (Essau *et al*., 2012; Ortuño-Sierra *et al*., 2015). The removal of the reverse worded items led to a common measurement model that showed an acceptable to good fit in each individual country. The finding that the reverse worded items had no significant or substantial relationships with their underlying latent factors might be explained by a methodological phenomenon called reversal ambiguity (Weijters and Baumgartner, 2012). Adolescents may not interpret the reverse worded items as opposites of the construct being measured and thus agree with both the reverse and positively worded items of the SDQ subscales. To illustrate, adolescents may agree with both the reverse worded item ‘I have one good friend or more’ *and* the positively worded item ‘Other children or young people pick on me or bully me’ of the peer relationship problems subscale. It is also possible that the reverse worded items tap into a different construct (e.g., Van de Looij-Jansen *et al*., 2011), and do not adequately measure a positive equivalence of mental health problems. Both these explanations substantiate our decision to remove the reverse worded items in order to establish the SDQ-R.

Notwithstanding the former, invariance tests indicated that the factor loadings of the *fights, lies, clingy, prefers adult, fidgety* and *distractible* items were non-invariant across all countries. Except for the fidgety item, these findings are in accordance with results from previous cross-country comparisons (Essau *et al*., 2012; Ortuño-Sierra *et al*., 2015). As such, partial measurement invariance is established, which means that latent means can still be compared across countries (Steinmetz, 2013). To facilitate the interpretation of latent mean differences we presented cross-country rankings.

Looking at the cross-country rankings found in this study, previous studies on cross-country variation in adolescents' *subjective well-being* found highly similar country rankings, with Greece and the Netherlands at the top and Poland at the bottom (Bradshaw and Richardson, 2009; Klocke *et al*., 2014; Inchley *et al*., 2016). Whereas a recent meta-analysis found no cross-country variation in adolescents' attention-deficit/hyperactivity disorders (ADHD) (Willcutt, 2012), this study found clear cross-country differences in adolescent self-reported hyperactivity-inattention problems. Interestingly, while Dutch adolescents reported the lowest levels of conduct problems and low levels of emotional symptoms and peer relationship problems, they reported by far the highest levels of hyperactivity-inattention problems. Future studies are encouraged to further investigate the found country differences in adolescent mental health problems.

In evaluating the SDQ-R some limitations should be considered. First, this study included data from different HBSC surveys. Although a recent trend analysis in the Netherlands based on the self-report SDQ revealed rather stable mental health problem levels over a 10-year period (Duinhof *et al*., 2015), we cannot exclude the possibility that our country rankings to some extent reflect time interval differences. Second, by removing the reverse worded items, the SDQ-R measures slightly different concepts of conduct problems, peer relationship problems and hyperactivity-inattention than the original self-report SDQ. To illustrate, the original hyperactivity-inattention subscale was designed to represent the three behavioural dimensions of a DSM-IV diagnoses of ADHD (American Psychiatric Association, 2013) and includes items measuring hyperactivity, inattention and impulsiveness (Goodman, 2001). By removing the reverse worded item from the hyperactivity-inattention problems subscale, the impulsiveness dimension of ADHD is not included in the SDQ-R anymore, and only one item taps into the inattention dimension. However, more generally, being a *brief* instrument for assessing adolescent mental health problems, one can debate whether the multidimensional nature of ADHD can be captured adequately by the SDQ at all (e.g., Garrido *et al*., 2018).

Third, the three-step method of invariance testing requires a referent indicator to identify the model (Muthén and Muthén, 2017), that is assumed to be perfectly invariant across groups. Non-invariant referent indicators may negatively impact the model fit and affect the results of invariance testing (Cheung and Rensvold, 1999; Johnson *et al*., 2009). A sensitivity analyses were conducted to make sure that the choice for the referent indicator did not influence the results negatively. For these, we ran several metric models by setting items consecutively as the referent indicator. The default setting (the first item as the referent indicator) showed one of the best model fits, and we continued with this metric model. Fourth, there is a debate about whether factor loadings and thresholds should be tested separately or in tandem to establish measurement invariance. We choose to test factor loadings and thresholds separately as this approach is less conservative and more explicit about the source of non-invariance (Bowen and Masa, 2015). Finally, CFA is known to produce inflated latent factor correlations if cross-loadings are meaningfully departing from zero in the population (Asparouhov and Muthén, 2009; Garrido *et al*., 2018). For example, in Romania, the MI suggested a cross-loading between the *distractible* item of the hyperactivity-inattention problems subscale and the peer relationship problems subscale. In CFA, such nonzero cross-loadings are fixed to zero, which may have been an overly stringent requirement for Romania, and resulted in overestimated latent factor intercorrelations. Thus, the latent factor correlations in this study need to be interpreted with care.

## Conclusion

Cross-country comparison using the SDQ have the great potential to advance our understanding of adolescent mental health. It can inform and drive global and national intervention and prevention efforts. The present study introduces a revised version of the self-report SDQ, the SDQ-R, that allowed for a valid comparison of adolescent mental health problems across seven countries of different regions in Europe. Mental health was relatively high in Greece, relatively low in Poland and most divergent in the Netherlands. To build our knowledge of adolescent mental health in- and outside Europe, future studies should further test the applicability of the SDQ-R, and further develop the self-report SDQ-R as a cross-country invariant measure of adolescent mental health problems.

## Availability of data and materials

Data of the HBSC study is available upon request. For more information, see http://www.hbsc.org.

## Appendix A

**Table A1.** Fit indices of the models testing for invariance between the 2005/2006 and 2013/2014 HBSC surveys in Bulgaria

Models	*χ*^2^	df	CFI	RMSEA	ΔCFI	ΔRMSEA	Model comparisons
1. Configural	6717.90*	328	0.835	0.064	–	–	–
2. Metric	6242.54*	344	0.848	0.060	0.013	−0.004	2 *v*. 1
3. Scalar	6237.68*	360	0.848	0.059	0.000	−0.001	3 *v*. 2

* = *p* < 0.001.

## Appendix B

**Table B1.** Items of the self-report SDQ in English and item abbreviations used in this study

Items in English		Item abbreviations
*Conduct problems*		
I get very angry and often lose my temper		Tempers
** I usually do as I am told**		**Obedient**
I fight a lot. I can make other people do what I want		Fights
I am often accused of lying or cheating		Lies
I take things that are not mine from home, school or elsewhere		Steals
*Emotional symptoms*		
I get a lot of headaches, stomach-aches or sickness		Somatic symptoms
I worry a lot		Worries
I am often unhappy, down-hearted or tearful		Unhappy
I am nervous in new situations. I easily lose confidence		Clingy
I have many fears, I am easily scared		Fears
*Peer relationship problems*		
I am usually on my own. I generally play alone or keep to myself		Solitary
** I have one good friend or more**		**Has good friend**
** Other people my age generally like me**		**Generally liked**
Other children or young people pick on me or bully me		Bullied
I get on better with adults than with people my own age		Prefers adult
*Hyperactivity-inattention problems*		
I am restless, I cannot stay still for long		Restless
I am constantly fidgeting or squirming		Fidgety
I am easily distracted, I find it difficult to concentrate		Distractible
** I think before I do things**		**Thinks before acting**
** I finish the work I'm doing. My attention is good**		**Good attention**

*Note.* Items in bold are the reverse worded items.

## Appendix C.

**Table C1.** Fit indices of the first-order factor models in the total sample

Models	*n*	*χ*^2^	df	CFI	RMSEA
First-order one-factor model	33 233	26 599.32*	170	0.748	0.068
First-order two-factor model	33 233	22 985.14*	169	0.782	0.064
First-order four-factor model	33 233	19 370.88*	164	0.817	0.059

* = *p* < 0.001.

## Appendix D.

**Table D1.** Fit indices of a first-order one-factor and first-order two-factor model based on the 15-item SDQ-R in the total sample and individual countries

	*n*	*χ*^2^	df	CFI	RMSEA
First-order one-factor model					
Total sample	33 220	12 872.72*	90	0.860	0.065
Bulgaria	4712	1727.31*	90	0.933	0.062
Germany	4991	3238.00*	90	0.735	0.084
Greece	4910	1659.40*	90	0.889	0.060
Netherlands	4241	3210.33*	90	0.780	0.090
Poland	5486	1930.83*	90	0.901	0.061
Romania	3886	1379.07*	90	0.869	0.061
Slovenia	4994	4397.22*	90	0.811	0.098
First-order two-factor model					
Total sample	33 220	9466.10*	89	0.897	0.056
Bulgaria	4712	1665.61*	89	0.936	0.061
Germany	4991	1808.02*	89	0.855	0.062
Greece	4910	1083.27*	89	0.930	0.048
Netherlands	4241	1744.87*	89	0.883	0.066
Poland	5486	1467.57*	89	0.925	0.053
Romania	3886	1237.90*	89	0.883	0.058
Slovenia	4994	3292.75*	89	0.860	0.085

* = *p* < 0.001.

## References

[ref1] Achenbach TM, Rescorla LA and Ivanova MY (2012) International epidemiology of child and adolescent psychopathology I: diagnoses, dimensions, and conceptual issues. Journal of the American Academy of Child and Adolescent Psychiatry 51, 1261–1272. 23200283 10.1016/j.jaac.2012.09.010

[ref2] American Psychiatric Association (2013) Diagnostic and Statistical Manual of Mental Disorders *(*DSM-*5^®^)*. Kernberg: American Psychiatric Association Publishing.

[ref3] Asparouhov T and Muthén BO (2009) Exploratory structural equation modeling. Structural Equation Modeling 16, 397–438.

[ref4] Bowen NK and Masa RD (2015) Conducting measurement invariance tests with ordinal data: a guide for social work researchers. Journal of the Society for Social Work and Research 6, 229–249.

[ref5] Bradshaw J and Richardson D (2009) An index of child well-being in Europe. Child Indicators Research 2, 319–351.

[ref6] Browne MW and Cudeck R (1992) Alternative ways of assessing model fit. Sociological Methods and Research 21, 230–258.

[ref7] Byrne BM (2012) Structural Equation Modeling with Mplus: Basic Concepts, Applications, and Programming. New York: Routledge.

[ref8] Chen FF (2007) Sensitivity of goodness of fit indexes to lack of measurement invariance. Structural Equation Modeling 14, 464–504.

[ref9] Cheung GW and Rensvold RB (1999) Testing factorial invariance across groups: a reconceptualization and proposed new method. Journal of Management 25, 1–27.

[ref10] Cheung GW and Rensvold RB (2002) Evaluating goodness-of-fit indexes for testing measurement invariance. Structural Equation Modeling 9, 233–255.

[ref11] Cohen J (1988) Statistical Power Analysis for the Behavioral Sciences, 2nd Edn. Hillsdale: Lawrence Erlbaum Associates.

[ref12] Currie C, Griebler R, Inchley J, Theunissen A, Molcho M, Samdal O and Dür W (2010) Health Behaviour in School-Aged Children (HBSC) Study Protocol: Background, Methodology and Mandatory Items for the 2009/2010 Survey. Edinburgh, Vienna: CAHRU, LBIHPR.

[ref13] Currie C, Inchley J, Molcho M, Lenzi M, Veselska Z and Wild F (2014) Health Behaviour in School-Aged Children (HBSC) Study Protocol: Background, Methodology and Mandatory Items for the 2013/2014 Survey. St Andrews: CAHRU.

[ref14] De Vries PJ, Davids EL, Mathews C and Aarø LE (2018) Measuring adolescent mental health around the globe: psychometric properties of the self-report strengths and difficulties questionnaire in South Africa, and comparison with UK, Australian and Chinese data. Epidemiology and Psychiatric Sciences 27, 369–380. 28112065 10.1017/S2045796016001207 PMC6998978

[ref15] Dimitrov DM (2010) Testing for factorial invariance in the context of construct validation. Measurement and Evaluation in Counseling and Development 43, 121–149.

[ref16] Duinhof EL, Stevens GWJM, van Dorsselaer S, Monshouwer K and Vollebergh WAM (2015) Ten-year trends in adolescents’ self-reported emotional and behavioral problems in the Netherlands. European Child and Adolescent Psychiatry 24, 1119–1128. 25534927 10.1007/s00787-014-0664-2

[ref17] Erskine HE, Baxter AJ, Patton G, Moffitt TE, Patel V, Whiteford HA and Scott JG (2017) The global coverage of prevalence data for mental disorders in children and adolescents. Epidemiology and Psychiatric Sciences 26, 395–402. 26786507 10.1017/S2045796015001158 PMC6998634

[ref18] Essau CA, Olaya B, Anastassiou-Hadjicharalambous X, Pauli G, Gilvarry C, Bray D, O'Callaghan J and Ollendick TH (2012) Psychometric properties of the strength and difficulties questionnaire from five European countries. International Journal of Methods in Psychiatric Research 21, 232–245. 22890628 10.1002/mpr.1364 PMC6878504

[ref19] Ford J, MacCallum R and Tait M (1986) The application of exploratory factor analysis in applied psychology: a critical review and analysis. Personnel Psychology 39, 291–314.

[ref20] Gadermann AM, Guhn M and Zumbo BD (2012) Estimating ordinal reliability for Likert-type and ordinal item response data: a conceptual, empirical, and practical guide. Practical Assessment, Research and Evaluation 17, 1–13.

[ref21] Garrido LE, Barrada JR, Aguasvivas JA, Martínez-Molina A, Arias VB, Golino HF, Legaz E, Ferris G and Rojo-Moreno L (2018) Is small still beautiful for the Strengths and Difficulties Questionnaire? Novel findings using exploratory structural equation modeling. Assessment 1–19. 10.1177/1073191118780461 29911418

[ref22] Goodman R (1997) The strengths and difficulties questionnaire: a research note. Journal of Child Psychology and Psychiatry 38, 581–586. 9255702 10.1111/j.1469-7610.1997.tb01545.x

[ref23] Goodman R (2001) Psychometric properties of the strengths and difficulties questionnaire. Journal of the American Academy of Child and Adolescent Psychiatry 40, 1337–1345. 11699809 10.1097/00004583-200111000-00015

[ref24] Hermida R (2015) The problem of allowing correlated errors in structural equation modeling: concerns and considerations. Computational Methods in Social Sciences 3, 5–17.

[ref25] Hu L and Bentler PM (1999) Cutoff criteria for fit indexes in covariance structure analysis: conventional criteria versus new alternatives. Structural Equation Modeling 6, 1–55.

[ref26] Inchley J, Currie D, Young T, Samdal O, Torsheim T, Augustson L, Mathison F, Aleman-Diaz A, Molcho M, Weber M and Barnekow V (2016) Growing up Unequal: Gender and Socioeconomic Differences in Young People's Health and Well-Being: Health Behaviour in School-Aged Children (HBSC) Study: International Report from the 2013/2014 Survey. Copenhagen: WHO Regional Office for Europe.

[ref27] Johnson EC, Meade AW and DuVernet AM (2009) The role of referent indicators in tests of measurement invariance. Structural Equation Modeling 16, 642–657.

[ref28] Klocke A, Clair A and Bradshaw J (2014) International variation in child subjective well-being. Child Indicators Research 7, 1–20.

[ref29] Kyriazos TA (2018) Applied psychometrics: the 3-faced construct validation method, a routine for evaluating a factor structure. Psychology 9, 2044–2072.

[ref30] Muthén LK and Muthén BO (2017) User's Guide, 8th Edn. Los Angeles: Muthén & Muthén.

[ref31] Nunnally J and Bernstein I (1967) Psychometric Theory. New York: McGraw-Hill.

[ref32] Ortuño-Sierra J, Fonseca-Pedrero E, Aritio-Solana R, Velasco AM, de Luis EC, Schumann G, Cattrell A, Flor H, Nees F, Banaschewski T, Bokde A, Whelan R, Buechel C, Bromberg U, Conrod P, Frouin V, Papadopoulos D, Gallinat J, Garavan H, Heinz A, Walter H, Struve M, Gowland P, Paus T, Poustka LM, Martinot J-L, Paillère-Martinot M-L, Vetter NC, Smolka MN and Lawrence C (2015) New evidence of factor structure and measurement invariance of the SDQ across five European nations. European Child and Adolescent Psychiatry 24, 1523–1534. 26036862 10.1007/s00787-015-0729-x

[ref33] Patel V, Flisher AJ, Hetrick S and McGorry P (2007) Mental health of young people: a global public-health challenge. Lancet 369, 1302–1313. 17434406 10.1016/S0140-6736(07)60368-7

[ref34] Polanczyk GV, Salum GA, Sugaya LS, Caye A and Rohde LA (2015) Annual research review: a meta-analysis of the worldwide prevalence of mental disorders in children and adolescents. Journal of Child Psychology and Psychiatry 56, 345–365. 25649325 10.1111/jcpp.12381

[ref35] Ravens-Sieberer U, Erhart M, Gosch A and Wille N (2008) Mental health of children and adolescents in 12 European countries - results from the European KIDSCREEN study. Clinical Psychology and Psychotherapy 15, 154–163. 19115436 10.1002/cpp.574

[ref36] Rutter M, Kim-Cohen J and Maughan B (2006) Continuities and discontinuities in psychopathology between childhood and adult life. Journal of Child Psychology and Psychiatry 47, 276–295. 16492260 10.1111/j.1469-7610.2006.01614.x

[ref37] Steinmetz H (2013) Analyzing observed composite differences across groups: is partial measurement invariance enough? Methodology 9, 1–12.

[ref38] Stevanovic D, Urbán R, Atilola O, Vostanis P, Singh Balhara YP, Avicenna M, Kandemir H, Knez R, Franic T and Petrov P (2015) Does the strengths and difficulties questionnaire-self report yield invariant measurements across different nations? Data from the International Child Mental Health Study Group. Epidemiology and Psychiatric Sciences 24, 323–334. 24785706 10.1017/S2045796014000201 PMC7192188

[ref39] Stevanovic D, Jafari P, Knez R, Franic T, Atilola O, Davidovic N, Bagheri Z and Lakic A (2017) Can we really use available scales for child and adolescent psychopathology across cultures? A systematic review of cross-cultural measurement invariance data. Transcultural Psychiatry 54, 125–152. 28157447 10.1177/1363461516689215

[ref40] Van de Looij-Jansen PM, Goedhart AW, de Wilde EJ and Treffers PD (2011) Confirmatory factor analysis and factorial invariance analysis of the adolescent self-report Strengths and Difficulties Questionnaire: how important are method effects and minor factors? British Journal of Clinical Psychology 50, 127–144. 10.1348/014466510X498174 21545447

[ref41] Vollebergh WA, Van Dorsselaer S, Monshouwer K, Verdurmen J, van der Ende J and ter Bogt T (2006) Mental health problems in early adolescents in the Netherlands. Social Psychiatry and Psychiatric Epidemiology 41, 156–163. 16453081 10.1007/s00127-005-0979-x

[ref42] Weijters B and Baumgartner H (2012) Misresponse to reversed and negated items in surveys: a review. Journal of Marketing Research 49, 737–747.

[ref43] Willcutt EG (2012) The prevalence of DSM-IV attention-deficit/hyperactivity disorder: a meta-analytic review. Neurotherapeutics 9, 490–499. 22976615 10.1007/s13311-012-0135-8 PMC3441936

[ref44] Yu C (2002) *Evaluating Cutoff Criteria of Model Fit Indices for Latent Variable Models with Binary and Continuous Outcomes* (Unpublished doctoral dissertation). University of California, Los Angeles.

